# Public Perceptions and Acceptance of COVID-19 Booster Vaccination in China: A Cross-Sectional Study

**DOI:** 10.3390/vaccines9121461

**Published:** 2021-12-10

**Authors:** Xiaozhen Lai, He Zhu, Jiahao Wang, Yingzhe Huang, Rize Jing, Yun Lyu, Haijun Zhang, Huangyufei Feng, Jia Guo, Hai Fang

**Affiliations:** 1 School of Public Health, Peking University, Beijing 100191, China; laixiaozhen@pku.edu.cn (X.L.); jiahaowang@pku.edu.cn (J.W.); yun.lyu@bjmu.edu.cn (Y.L.); haijunzhang@pku.edu.cn (H.Z.); yffenghuang@pku.edu.cn (H.F.); 1610306212@pku.edu.cn (J.G.); 2 China Center for Health Development Studies, Peking University, Beijing 100191, China; he.zhu@pku.edu.cn; 3 Department of Statistics and Modeling, Beijing Weikexing Technology, Beijing 100191, China; yingzhe_huang@hotmail.com; 4 School of Public Administration and Policy, Renmin University of China, Beijing 100872, China; rzjing@ruc.edu.cn; 5 Peking University Health Science Center, Chinese Center for Disease Control and Prevention, Joint Center for Vaccine Economics, Beijing 100191, China; 6 Key Laboratory of Reproductive Health, National Health Commission of the People’s Republic of China, Beijing 100191, China

**Keywords:** booster vaccination, China, COVID-19, health belief model, vaccine

## Abstract

Coronavirus disease 2019 (COVID-19) booster vaccination has been proposed in response to the new challenges of highly contagious variants, yet few studies have examined public acceptance of boosters. This study examined public acceptance of COVID-19 booster vaccination and its influencing factors by using the data from a self-administered online cross-sectional survey conducted in June 2021 in China. Multiple logistic analysis was used to examine the influencing factors of booster acceptance based on the health belief model (HBM). Among 1145 respondents, 84.80% reported to accept COVID-19 booster vaccination. Having COVID-19 vaccination history, perceiving high benefits and low barriers to booster vaccination, being younger (18–30 vs. 41–50), having a lower education level, being employed, and belonging to priority groups for vaccination were associated with increased odds of booster acceptance. The primary reason for refusing booster vaccination was concern about vaccine safety. The vast majority (92.8%) of respondents reported an annual willingness to pay between 0 and 300 CNY (0–46.29 USD) if the booster was not free. Our findings suggest that the acceptance rate of booster vaccination is relatively high in China, and the HBM-based analysis reveals that more efforts are needed to increase perceived benefits and reduce perceived barriers of vaccination to design effective and proper vaccination extension strategies when boosters become widely recommended.

## 1. Introduction

Coronavirus disease 2019 (COVID-19) booster vaccination has been proposed and is undergoing experiments or pilots in response to the new challenges of COVID-19 brought by highly contagious variants [1]. A striking example of such a COVID-19 variant is the B.1.617.2 (delta) variant, which has led to a surge in infection cases across the globe [2]. Moreover, the effectiveness of approved COVID-19 vaccines against variants remains unclear. One recent study reported that the effectiveness of two-dose BNT162b2 (mRNA) and ChAdOx1 (adenovirus vector) vaccines remained as high as 88% and 67%, respectively, among patients with the Delta variant [3]. Another laboratory study found that the antibodies produced by mRNA vaccines still offered protection against the B.1.1.7 (alpha) and B.1.351 (beta) variants, but the protection was much less strong [2]. Until May 2021, three inactivated vaccines, one adenovirus vector vaccine, and one recombinant subunit vaccine against COVID-19 have been used in China, and all of them were domestically produced [4]. Among them, inactivated vaccines possessed the largest market share and were the most frequently administered [5]. An Indian case-control study published in November 2021 showed that the adjusted effectiveness of the two-dose BBV152 (inactivated vaccine) at least 14 days before testing was 47% and suggested that the relatively low effectiveness might be due to the high prevalence of the circulating delta variant in India [6]. Infectious disease experts have carefully weighed the need for booster shots for certain vulnerable groups or the entire population to protect against the circulating new variants and improve immunity level, as the duration of protection remains unknown [7]. As concerns about variants and protection duration continue to alarm the public of the importance of booster shots, it is imperative to make some preparations in advance to understand the demands for booster vaccination.

The World Health Organization (WHO) has also considered annual boosters for high-risk individuals and boosters every two years for the general population [8]. An increasing number of countries are now delivering a third booster shot for the public, and Israel took the initiative to supply boosters for the elderly aged over 60 years on 30 July 2021 [9]. In China, the mass immunization program has been progressing smoothly since its launch on 31 December 2020 [10], and the country has delivered 2.47 billion vaccine doses as of 27 November 2021 [11]. Clinical evidence supports the widely acknowledged two-dose vaccination schedule [12], but limited clinical evidence is available to support the necessity of boosters in China [13]. However, it was reported in late September 2021 that China would launch COVID-19 booster vaccinations for priority populations aged 18–59 years old who had completed two-dose immunization six months before. Later, local areas such as Zhejiang and Henan provinces officially announced the implementation of booster vaccination programs, which could soon be launched throughout the country.

Although there have been many studies examining public acceptance of current COVID-19 vaccination showing that acceptance varied substantially globally [14,15,16], little is known about booster acceptance in China. A declining trend of COVID-19 vaccination acceptance rates has been found in China [17] and the United States [18,19], and it has been noted that many factors could be considered in the interpretation of varied vaccine acceptance in different countries [14,20,21,22,23]. Another issue with the blooming controversy is whether the COVID-19 vaccine needs annual (or regular) boosters to maintain high levels of immunity against both the original virus and variants, similar to annual seasonal influenza shots. Therefore, there is an urgent need to understand the public acceptance of COVID-19 boosters to prepare for effective promotion strategies [14,15].

To fill the gap in booster vaccination acceptance in China, this study aims to examine the public acceptance rate of COVID-19 boosters and its influencing factors using a self-administered online survey conducted in June 2021. This study also attempts to explore the willingness to pay (WTP) for annual boosters in case of yearly surges of the COVID-19 pandemic. The study of booster acceptance will help provide empirical evidence to improve COVID-19 booster delivery.

## 2. Materials and Methods

### 2.1. Study Design and Study Sample

A web-based anonymous cross-sectional survey was conducted to measure public acceptance of COVID-19 booster vaccination during 3–21 June 2021 in China as a follow-up survey of public acceptance of COVID-19 vaccines [17,24]. Details of the design and conduct of the survey have been previously described [17,24]. Briefly, the survey was launched on the largest online survey platform, Wen Juan Xing (Changsha Ranxing Information Technology Co., Ltd., Changsha, China), which consists of over 2.6 million Chinese members with confirmed personal information and diverse socioeconomic backgrounds, and allows automatic logical proofreading to reduce input errors and avoid missing values [24]. The target population of the survey was Chinese adults living in the mainland of China, and a sampling method stratified by age and location was adopted to match the characteristics of the Chinese adult population in the Wen Juan Xing sample database. A total of 3280 adults were invited to participate in the survey via Wen Juan Xing, and 1167 completed the survey with a response rate of 35.58%. We limited our study sample to respondents aged 18–59 years old because older adults often have difficulties accessing online surveys. Then, the final sample was 1145 adults aged 18–59 years old (22 aged 60 and above were excluded from the 1167 survey participants). This study was ethically reviewed and approved by the Peking University Institutional Review Board (IRB 00001052-20011).

The structured online questionnaire was designed based on previous studies on vaccine acceptance [25,26,27,28], and it included items regarding sociodemographic characteristics, self-perceived health status, COVID-19 vaccination history, perceptions of COVID-19 infection and booster vaccination, intention to receive a COVID-19 booster, and WTP to receive annual COVID-19 boosters.

### 2.2. Conceptual Framework

We used the health belief model (HBM), developed in the 1950s, as a conceptual framework to understand the acceptance of COVID-19 boosters, which has been widely used to understand the determinants of vaccination intentions [29,30,31]. The HBM considers that positive factors could increase pro-health behaviors while negative factors could decrease or inhibit them. The HBM model comprises six dimensions (i.e., five positive aspects and one negative aspect) [32,33,34]. For a patient in need of certain health-related behaviors, positive aspects include (1) perceived susceptibility: believing to be susceptible to the disease; (2) perceived severity: believing that the disease will negatively impact quality of life; (3) perceived benefits: believing that adopting the behaviors is beneficial to reduce susceptibility or severity; (4) self-efficacy: having the capacity to engage in the behaviors; and (5) cues to action: having motivations to adopt the behaviors. The negative aspect refers to perceived barriers: believing that there are some restrictions or obstructions when adopting the behaviors [32,33,34].

Specifically for applying the HBM model to infectious diseases and vaccination, perceived susceptibility refers to feelings regarding personal vulnerability to the infectious disease; perceived severity refers to beliefs regarding the negative effects of the infectious disease; perceived benefits focus on the effectiveness of vaccination in reducing disease susceptibility or severity; perceived barriers represent the issues that potentially restrict individuals from vaccination; self-efficacy accounts for individual capacity to engage in vaccination; and cues to action refer to internal or external stimuli or information that spurs an individual to get vaccinated [34,35,36].

### 2.3. Measures

#### 2.3.1. COVID-19 Booster Acceptance

The primary outcome was the acceptance of COVID-19 booster vaccination, which was defined based on the following question: “If a COVID-19 booster is recommended as a supplement to the current vaccination schedule, would you accept it? (Yes/No)” (see Table A1 in Appendix A). Respondents were classified into the booster acceptance group or rejection group accordingly. Then, respondents in the booster acceptance group were asked for reasons of acceptance, and those in the rejection group were asked for reasons of rejection. In addition, WTP was measured by using a one-item open-ended question: “What is the maximum amount that you are willing to pay for an annual COVID-19 booster vaccination?” [37].

#### 2.3.2. History and Priority Groups for COVID-19 Vaccination

Respondents’ COVID-19 vaccination history was collected from the question: “Have you received at least one dose of COVID-19 vaccine so far? (Yes/No)”. Priority groups for COVID-19 vaccination were defined as persons who were vaccination targets with higher infection risk in China, such as health professionals, community workers, workers in the cold-chain logistics sector, and customs inspectors [38].

#### 2.3.3. Perceptions of COVID-19 Infection and Booster Vaccination

In this study, HBM items were designed to measure perceptions of COVID-19 infection and booster vaccination: perceived susceptibility to COVID-19 infection (two questions), perceived severity of COVID-19 infection (two questions), perceived benefits of COVID-19 boosters (three questions), perceived barriers to receiving COVID-19 booster vaccination (two questions), self-efficacy to engage in vaccination (one question), and cues to action (two questions). The detailed questions can be found in Table A2 (Appendix A). All self-reported response options were assessed on a five-point Likert scale and converted to binary variables [20,29], e.g., agree (i.e., “strongly agree” or “agree”) and neutral/disagree (i.e., “neither agree nor disagree”, “disagree”, or “strongly disagree”).

#### 2.3.4. Sociodemographic Characteristics

Sociodemographic characteristics included age groups (18–30, 31–40, 41–50, and 51–59 years old), gender (male and female), maternal status (married and never married/divorced/widowed), education level (senior high school/technical school or below and college/associate/bachelor’s degree or above), employment status (employed and retired/out of work/still a student), household annual income (≤100,000, 100,001–200,000, and >200,000 CNY), residence (urban and rural), and region (western, central, and eastern). The respondents were also asked to rate their own overall health status and report whether they had any chronic disease. All questions were closed-ended and treated as categorical variables.

### 2.4. Statistical Analysis

Descriptive statistics were performed to describe the characteristics of the study sample, as well as the distribution of perceptions and acceptance of COVID-19 booster vaccination. We produced summary statistics using frequencies and proportions for categorical variables, and the chi-square test was used to examine differences between COVID-19 perceptions and COVID-19 booster acceptance. Based on the HBM model, we conducted unadjusted analyses followed by a multiple logistic regression analysis to examine associations of perceptions, vaccination history, and sociodemographics with COVID-19 booster vaccination acceptance. A two-sided *p* value below 0.05 was considered statistically significant. Additionally, bar diagrams were used to depict the characteristics of reasons for accepting or not accepting booster vaccination. Finally, the distribution of annual WTP for booster vaccination was examined. All data were analyzed using Stata 14.0 (StataCorp, College Station, TX, USA).

## 3. Results

### 3.1. Characteristics of the Study Sample

As shown in Table 1, of the total sample (*n* = 1145), nearly two-thirds (65.68%) were aged 18–40 years old, 50.31% were female, 79.13% were married, and 72.84% had an education level of college/associate/bachelor’s degree or above. The majority of the respondents were employed (87.34%), had a total household annual income ranging from 100,001 to 200,000 CNY (15,430 to 30,860 USD) (46.2%), resided in urban areas (88.47%), and were located in the eastern region of China (66.29%). Regarding health status, 71.62% reported good health and 12.75% reported to have any chronic disease. Additionally, 21.92% belonged to priority groups for vaccination, and 79.30% had received at least one dose of a COVID-19 vaccine by June 2021.

Overall, 84.80% of respondents reported to accept COVID-19 booster vaccination, and the booster acceptance rate varied across different sociodemographic groups. Respondents belonging to priority groups for vaccination (90.84%) and having previous vaccination history against COVID-19 (89.21%) had relatively high booster vaccination acceptance rates. Conversely, those aged 41–50 years old (79.23%), being retired/out of work/still a student (74.48%), with fair or poor health status (78.77%), with any chronic disease (78.08%), and without COVID-19 vaccination history (67.93%) had relatively low booster acceptance rates.

### 3.2. HBM Factors and Booster Vaccination Acceptance

In terms of HBM factors (Table 2), the majority of respondents perceived a fair or low risk of COVID-19 infection (91.70%) and a higher risk of COVID-19 infection from a variant than from existing strains (85.41%); agreed with a high severity of COVID-19 infection (72.49%) and more severe illness caused by variants compared with existing strains (82.36%); believed in the booster’s efficacy against early circulating strains (84.02%), to extend protection (81.83%), and against variants (76.33%); perceived high safety of COVID-19 boosters (83.67%); were worried about serious adverse reactions less (82.01%); agreed that it would be easy to get the COVID-19 vaccine if wanted (73.8%); did not have confirmed or suspected cases in daily close contacts (94.50%); and knew about at least one foreign variant (85.94%).

Chi-square tests indicated that there were significant differences between the HBM dimensions and booster acceptance except for two factors: the risk of COVID-19 infection in perceived susceptibility and confirmed or suspected case contacts in cues to action. More specifically, respondents’ booster vaccination acceptance was positively correlated with perceived susceptibility, severity, benefits, self-efficacy, and cues to action, while the correlation was negative between booster acceptance and worrying about serious adverse reaction after vaccination.

### 3.3. Adjusted Analysis of Booster Acceptance

Multiple logistic regression was performed after unadjusted models to identify the influencing factors of booster acceptance (Table 3). Previous vaccination history against COVID-19 (adjusted odds ratio [aOR]: 3.05, 95% confidence interval [CI]: 2.05–4.54) was a strong predictor of booster vaccination acceptance. For HBM factors, those perceiving a high efficacy of boosters against early circulating strains (aOR: 1.86, 95% CI: 1.11–3.13) and perceiving a high efficacy of boosters to extend protection (aOR: 1.64, 95% CI: 1.04–2.61) were more likely to accept COVID-19 booster vaccination. In contrast, those perceiving a lower safety of COVID-19 boosters (aOR: 0.21, 95% CI: 0.13–0.35) and those worried about serious adverse reactions after vaccination (aOR: 0.63, 95% CI: 0.41–0.97) were less likely to accept booster vaccination. In addition, respondents aged 41–50 (vs. 18–30), with a college/associate/bachelor’s degree or above (vs. senior high school/technical school or below), being retired/out of work/still a student (vs. employed), and not belonging to a priority vaccination group (vs. priority groups) were associated with decreased odds of booster vaccination acceptance.

### 3.4. Reasons for Accepting or Not Accepting Booster Vaccination

**Figure 1:**
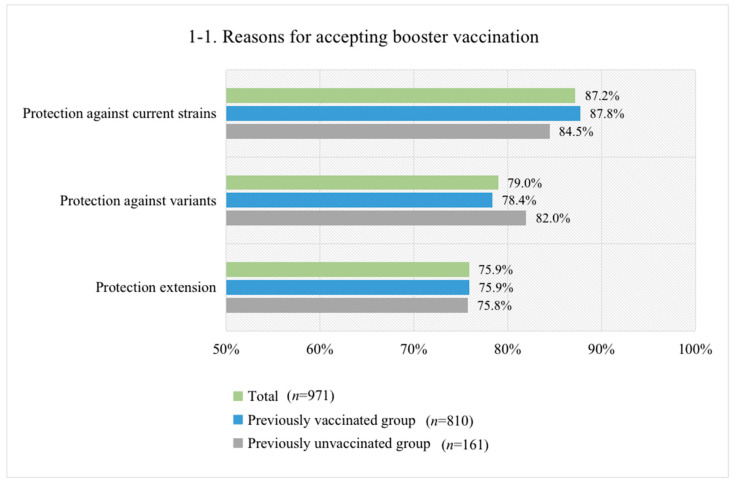

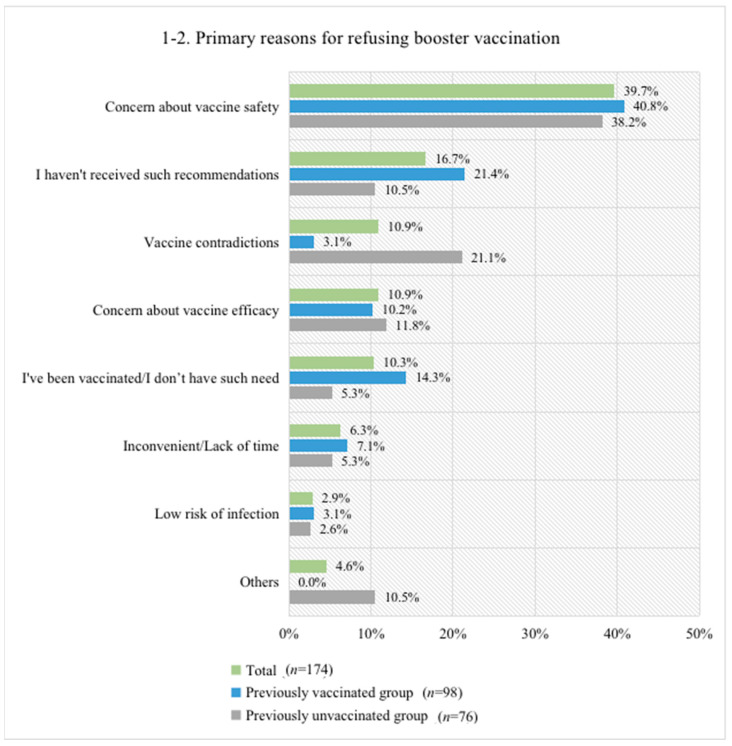
Reasons for accepting (**1-1**) or refusing (**1-2**) booster vaccination. Note: The reasons for accepting booster vaccination were not mutually exclusive, and the percentage sum of all the reasons was more than 100%, as some respondents chose more than one reason. In contrast, primary reasons for refusing booster vaccination were mutually exclusive, and the percentage sum of all the reasons was 100%.

The primary reason for accepting booster vaccination was “protection against current strains” (i.e., early circulating strains) for both previously vaccinated respondents (87.8%) and unvaccinated respondents (84.5%) (Figure 1(1-1)). Other reasons for acceptance included “protection against variants” (79.0%) and “protection extension” (75.9%). The most common reason for not accepting booster vaccination was “concern about vaccine safety” for both the vaccinated (40.8%) and unvaccinated (38.2%) groups (Figure 1(1-2)). Other common rejection reasons included “I haven’t received such recommendations” (21.4%), “I’ve been vaccinated/I don’t have such need” (14.3%) and “concern about vaccine efficacy” (10.2%) for the previously vaccinated group, and “vaccine contradictions” (21.1%), “concern about vaccine efficacy” (11.8%), and “I haven’t received such recommendations” (10.5%) for the previously unvaccinated group.

### 3.5. Willingness to Pay (WTP) for an Annual Booster Vaccination

Among the 1063 respondents (92.84% of the total sample) who provided answers for their WTP for an annual booster vaccination, the mean and median WTP were 118.6 CNY and 60.0 CNY, respectively (Table 4). Approximately 32.7%, 17.6%, and 13.3% of respondents reported that they would pay zero, 100 CNY, and 200 CNY, respectively. Cumulatively, among the non-refusers, 92.8% of respondents were willing to pay an annual fee between 0 and 300 CNY, and 7.2% were willing to pay an annual fee of 301 CNY or above.

## 4. Discussion

To our knowledge, this is the first study to investigate public acceptance of COVID-19 booster vaccination in China, and it is prudent to prepare for such a need as a precaution. We found that 84.80% of respondents aged 18–59 years old reported their willingness to accept COVID-19 booster vaccination, and perceived benefits and barriers were two important HBM dimensions associated with booster acceptance. The primary reasons for accepting or not accepting booster vaccination were “protection against current strains” and “concern about vaccine safety”, respectively. Additionally, over 90% reported WTP for an annual booster vaccination between 0 and 300 CNY (0–46.29 USD). Our findings have important implications for effective and proper interventions for future booster vaccination campaigns against COVID-19 and its variants.

This study was conducted approximately six months after the implementation of national COVID-19 vaccination programs in China, when new variants, especially the delta variant, appeared to threaten global public health [11]. Our findings indicated that a large proportion of respondents (84.80%) expressed their intention to receive COVID-19 booster vaccinations. At present, few studies have been published on booster acceptance, and it is difficult to make direct domestic or inter-country comparisons. We compared our findings of booster acceptance rates with the public acceptance of non-booster COVID-19 vaccine acceptance rates, and found that generally, the booster acceptance rate was close to some non-booster vaccine acceptance rates reported in China, such as the 88.5% shown in a longitudinal study in the well-contained phase (Nov–Dec 2020) [17]. However, the booster acceptance rate in this study was much higher than the non-booster vaccine acceptance rate (55.3%) reported by Zhao et al. in Jan–Apr 2021 [39]. The higher acceptance rate of boosters reported in this study may be partly explained by higher public expectations of boosters facing the new challenges of variants [11], as well as the observed increasing trend in COVID-19 vaccination acceptance in the first half of 2021 [39] since this study was conducted in June 2021, several months after Zhao et al.’s survey.

Based on HBM, our results suggested that perceived benefits and perceived barriers to vaccination were important dimensions associated with the acceptance of COVID-19 boosters, which is consistent with the HBM-related findings of the existing H1N1 and COVID-19 vaccination studies [29]. Vaccine efficacy has been reported as an important predictor of vaccine acceptance and uptake [15,30,40], and a discrete choice experiment (DCE) study conducted in China found that high effectiveness of COVID-19 vaccines was the most favored attribute [41]. The importance of perceived barriers has likewise been reported in previous studies of COVID-19 vaccines [15,42], and it was emphasized that strict surveillance during booster development should be organized. It is important to improve health promotion and reduce barriers to booster vaccination. Hence, public health intervention programs should focus on increasing beliefs about vaccine effectiveness and reducing perceived adverse effects and safety barriers if boosters are widely approved for mass vaccination against COVID-19 variants in the future.

Our findings also indicated that COVID-19 vaccination history was strongly associated with increased odds of accepting COVID-19 booster vaccination. Prior studies also found that vaccination history had a positive effect on individual vaccination intention [27,43,44]. For example, one study in Australia found that the acceptance of previous influenza vaccination had the largest effect on the willingness to be vaccinated against H1N1 during the 2009 pandemic (OR = 5.03) [27]. Previous vaccination history should be carefully considered when designing vaccination schedules and targeted measures. The multiple logistic regression of sociodemographic characteristics indicated that respondents aged 41–50 and with a higher education level expressed significantly lower booster acceptance, while employment or belonging to priority groups for vaccination remained indicators of higher booster acceptance. These results were inconsistent with previous studies in China concerning COVID-19 vaccine acceptance that highlighted the impacts of gender, marital status, and regional or rural/urban differences [17,20,29]. Further studies are needed to investigate the differences in sociodemographic characteristics between vaccine acceptance and booster acceptance. The associations between sociodemographic characteristics and booster acceptance in this study could provide preliminary results to help the design of booster-targeted vaccination strategies to increase coverage.

The reasons for accepting or not accepting booster vaccination were further explored, with protection against early circulating strains being the primary reason for booster acceptance, and safety concerns being the priority issue for refusers. Public concern about vaccine safety continues to be an obvious obstacle for booster uptake, and the top priority should be to strengthen public trust in both vaccination and booster safety by adhering to post-marketing surveillance and improving the compensation policy after adverse events [45,46,47]. For booster refusers, there were also differences between previously vaccinated and unvaccinated groups. The previously vaccinated group was more concerned about vaccine recommendations or the need to be vaccinated again, while the unvaccinated group posed more concerns about contradictions and vaccine efficacy. Therefore, for previously vaccinated respondents, more efforts could be made concerning recommendations from reliable information sources and emphasis on the need for and importance of boosters; for previously unvaccinated respondents, more attention could be paid to the correct understanding of vaccine contradictions and the effectiveness of booster shots.

Vaccine price may be an important obstacle for the acceptance of self-paid vaccines [48,49]. The Chinese government is providing COVID-19 vaccination free of charge to the general public, but it is unknown whether this policy will be maintained. Our findings revealed that the mean and median WTP for annual booster vaccination were 118.62 CNY (18.30 USD) and 60 CNY (9.26 USD), respectively, which was lower than those reported for full-course COVID-19 vaccination when vaccines were not yet available (mean: 254 CNY (39.19 USD); median: 100 CNY (15.43 USD)) [24]. This decline may be explained by the annual requirement and currently free vaccination. The purpose of exploring WTP is not to encourage the transition from free vaccination to out-of-pocket or partially out-of-pocket payment, but to provide a reference for policy makers to make decisions on future vaccination policies.

There are several limitations in this study. First, due to the intrinsic disadvantages of cross-sectional online surveys, sampling bias may exist to limit the representativeness of the results [50,51]. This study tried to reduce bias by recruiting adults using a stratified sampling method. Second, self-reported responses may be subjective to recalling bias and a tendency to report socially desirable responses. We designed an anonymous survey with most questions asking respondents’ thoughts and feelings at the moment, which may have helped to minimize the effect of self-reporting bias. Third, booster acceptance and WTP were derived based on hypothetical COVID-19 booster vaccination before the approval of final products [52]. Future research to gather more accurate acceptance of COVID-19 boosters is encouraged to prepare for the booster campaign.

## 5. Conclusions

In conclusion, our findings preliminarily indicate a relatively large proportion of respondents accepting COVID-19 booster vaccination in China. The HBM-based analysis reveals that more efforts are needed to increase perceived benefits and reduce perceived barriers of vaccination to enhance the acceptance of COVID-19 boosters, whereas perceived susceptibility, perceived severity, self-efficacy, and cues to action have relatively low predictive power. Our results could serve as a reference for China and other countries in analyzing public perceptions and acceptance of COVID-19 booster vaccination to design effective and proper vaccination extension strategies when boosters become widely recommended in the future.

## Figures and Tables

**Table 1:** Sociodemographic characteristics of the study sample and the proportions of booster vaccination acceptance.

	Total		Booster Vaccination Acceptance
	*n*	Column %	Row %
Total	1145	100.00	84.80
Age group, years			
18–30	349	30.48	87.11
31–40	403	35.20	86.85
41–50	284	24.80	79.23
51–59	109	9.52	84.40
Gender			
Male	569	49.69	86.64
Female	576	50.31	82.99
Maternal status			
Married	906	79.13	85.32
Never married/divorced/widowed	239	20.87	82.85
Education level			
Senior high school/technical school or below	311	27.16	86.82
College/associate/bachelor’s degree or above	834	72.84	84.05
Employment status			
Employed	1000	87.34	86.30
Retired/out of work/still a student	145	12.66	74.48
Household annual income			
≤100,000 CNY	352	30.74	84.09
100,001–200,000 CNY	529	46.20	84.69
>200,000 CNY	264	23.06	85.98
Residence			
Urban	1013	88.47	84.60
Rural	132	11.53	86.36
Region			
Eastern	759	66.29	83.53
Central	233	20.35	87.55
Western	153	13.36	86.93
Self-reported health status			
Good	820	71.62	87.20
Fair/poor	325	28.38	78.77
Having any chronic disease			
Yes	146	12.75	78.08
No	999	87.25	85.79
Priority groups for vaccination			
Yes	251	21.92	90.84
No	894	78.08	83.11
Received COVID-19 vaccination			
Yes	908	79.30	89.21
No	237	20.70	67.93

Note: CNY: Chinese Yuan, 1 CNY = 0.1543 USD on 25 July 2021.

**Table 2:** Perceptions of vaccination and booster vaccination acceptance based on the health belief model.

Factors	Total		Booster Vaccination Accept		Booster Vaccination Refuse		*p* Value
	*n*	Column %	*n*	Column %	*n*	Column %	
**Perceived susceptibility**							
The risk of COVID-19 infection							0.668
High	95	8.30	82	8.44	13	7.47	
Fair/low	1050	91.70	889	91.56	161	92.53	
Variants have higher risk of infection than the existing strains							<0.001
Agree	978	85.41	849	87.44	129	74.14	
Neutral/disagree	167	14.59	122	12.56	45	25.86	
**Perceived severity**							
The severity of COVID-19 infection							0.040
High	830	72.49	715	73.64	115	66.09	
Fair/low	315	27.51	256	26.36	59	33.91	
Variants can cause more severe illness than the existing strains							<0.001
Agree	943	82.36	822	84.65	121	69.54	
Neutral/disagree	202	17.64	149	15.35	53	30.46	
**Perceived benefits**							
Efficacy of boosters against early circulating strains							<0.001
High	962	84.02	854	87.95	108	62.07	
Fair/low	183	15.98	117	12.05	66	37.93	
Efficacy of boosters to extend protection							<0.001
High	937	81.83	829	85.38	108	62.07	
Fair/low	208	18.17	142	14.62	66	37.93	
Efficacy of boosters against variants							<0.001
High	874	76.33	782	80.54	92	52.87	
Fair/low	271	23.67	189	19.46	82	47.13	
**Perceived barriers**							
Safety of boosters							<0.001
High	958	83.67	867	89.29	91	52.30	
Fair/low	187	16.33	104	10.71	83	47.70	
Worry about serious adverse reaction after vaccination							<0.001
High	206	17.99	152	15.65	54	31.03	
Fair/low	939	82.01	819	84.35	120	68.97	
**Self-efficacy**							
It is easy to get the COVID-19 vaccine if wanted							<0.001
Agree	845	73.80	744	76.62	101	58.05	
Neutral/disagree	300	26.20	227	23.38	73	41.95	
**Cues to action**							
Used to have confirmed or suspected cases in daily close contacts							0.353
Yes	63	5.50	56	5.77	7	4.02	
No	1082	94.50	915	94.23	167	95.98	
Know about at least one foreign variant							0.003
Yes	984	85.94	847	87.23	137	78.74	
No	161	14.06	124	12.77	37	21.26	

**Table 3:** Unadjusted and adjusted logistic regression analyses of factors of booster vaccination acceptance.

Factors	Unadjusted Logistic Model		Adjusted Logistic Model ^†^	
	Crude OR	95% CI	Adjusted OR (aOR)	95% CI
Received COVID-19 vaccination (yes vs. no)	3.90 *	(2.77, 5.50)	3.05 *	(2.05, 4.54)
**Perceived susceptibility**				
High risk of COVID-19 infection (yes vs. no)	1.14	(0.62, 2.10)	1.00	(0.48, 2.08)
Variants have higher risk of infection than the existing strains (yes vs. no)	2.43 *	(1.65, 3.58)	1.03	(0.59, 1.80)
**Perceived severity**				
High severity of COVID-19 infection (yes vs. no)	1.43 *	(1.02, 2.02)	0.93	(0.60, 1.43)
Variants can cause more severe illness than the existing strains (yes vs. no)	2.42 *	(1.67, 3.49)	1.14	(0.67, 1.96)
**Perceived benefits**				
High efficacy of boosters against early circulating strains (yes vs. no)	4.46 *	(3.11, 6.41)	1.86 *	(1.11, 3.13)
High efficacy of boosters to extend protection (yes vs. no)	3.57 *	(2.50, 5.09)	1.64 *	(1.04, 2.61)
High efficacy of boosters against variants (yes vs. no)	3.69 *	(2.63, 5.17)	1.30	(0.81, 2.10)
**Perceived barriers**				
Low safety of boosters (yes vs. no)	0.13 *	(0.09, 0.19)	0.21 *	(0.13, 0.35)
Worry about serious adverse reaction after vaccination (yes vs. no)	0.41 *	(0.29, 0.59)	0.63 *	(0.41, 0.97)
**Self-efficacy**				
It is easy to get the COVID-19 vaccine if wanted (yes vs. no)	2.37 *	(1.69, 3.32)	1.24	(0.79, 1.93)
**Cues to action**				
Used to have confirmed or suspected cases in daily close contacts (yes vs. no)	1.46	(0.65, 3.26)	2.77	(0.98, 7.82)
Know about at least one foreign variant (yes vs. no)	1.85 *	(1.23, 2.78)	1.20	(0.70, 2.06)
**Sociodemographics**				
Age group, years (vs. 18–30)				
31–40	0.98	(0.64, 1.50)	0.91	(0.51, 1.60)
41–50	0.57 *	(0.37, 0.86)	0.52 *	(0.29, 0.91)
51–59	0.80	(0.44, 1.47)	0.97	(0.46, 2.04)
Female (vs. male)	0.75	(0.54, 1.04)	0.76	(0.52, 1.10)
Married (vs. never married/divorced/widowed)	1.20	(0.82, 1.77)	1.01	(0.58, 1.75)
College/associate/bachelor’s degree or above (vs. senior high school/technical school or below)	0.80	(0.55, 1.17)	0.49 *	(0.30, 0.81)
Employed (vs. retired/out of work/still a student)	2.16 *	(1.43, 3.27)	1.84 *	(1.06, 3.20)
Household annual income (vs. ≤100,000 CNY)				
100,001–200,000 CNY	1.05	(0.72, 1.52)	0.92	(0.58, 1.45)
>200,000 CNY	1.16	(0.74, 1.82)	1.16	(0.64, 2.09)
Residing in urban areas (vs. rural)	0.87	(0.51, 1.47)	0.81	(0.42, 1.55)
Region (vs. eastern)				
Central	1.39	(0.90, 2.14)	1.26	(0.76, 2.10)
Western	1.31	(0.79, 2.18)	1.71	(0.96, 3.06)
Self-reported health status (good vs. fair/poor)	1.84 *	(1.31, 2.57)	0.80	(0.53, 1.23)
Having any chronic disease (yes vs. no)	0.59 *	(0.38, 0.91)	0.60	(0.33, 1.06)
Belonging to priority groups for vaccination (yes vs. no)	2.02 *	(1.27, 3.20)	1.98 *	(1.13, 3.46)

Notes: * *p* < 0.05. OR: odds ratio. CI: confidence interval. CNY: Chinese Yuan, 1 CNY = 0.1543 USD on 25 July 2021. ^†^ Goodness of fit: Pearson chi-square = 1030.26, Prob > chi2 = 0.2261.

**Table 4:** The distribution of expected annual WTP (CNY) for COVID-19 booster vaccination.

WTP Value (CNY) ^a^	*n*	Percent (%)	Cumulative Percent (%)	Cumulative Percent of Non-Refusers (%)
Refusing to vaccinate boosters and report WTP	82	7.2	7.2	-
0	374	32.7	39.8	35.2
1~49	59	5.2	45.0	40.7
50	95	8.3	53.3	49.7
51~99	26	2.3	55.5	52.1
100	201	17.6	73.1	71.0
101~199	27	2.4	75.5	73.6
200	152	13.3	88.7	87.9
201~299	3	0.3	89.0	88.1
300	49	4.3	93.3	92.8
301~499	17	1.5	94.8	94.4
500	34	3.0	97.7	97.6
501~999	14	1.2	99.0	98.9
1000	10	0.9	99.8	99.8
1001~3000	2	0.2	100.0	100.0
Mean of WTP	118.62 CNY			
Median of WTP	60 CNY			

Note: CNY: Chinese Yuan, 1 CNY = 0.1543 USD on 25 July 2021. ^a^ Willingness to pay (WTP) values were measured using a one-item open-ended question (What is the maximum amount you are willing to pay for an annual COVID-19 booster vaccination?).

## Data Availability

The data presented in this study are available on request from the corresponding author.

## Appendix A

**Table A1:** Question list of key variables used in this survey.

Measures	Questions	Question Design and Coding
**COVID-19 booster acceptance**	If a COVID-19 booster is recommended as a supplement to the current vaccination schedule, would you accept it?	Binary (“yes [1]” or “no [0]”).
	What are the reasons for accepting booster vaccination? What is the primary reason for refusing booster vaccination?	Multiple choices for question 1, and single choice for question 2.
**Willingness to pay**	What is the maximum amount that you are willing to pay for an annual COVID-19 booster vaccination?	Open-ended question with values.
**History of COVID-19 vaccination**	Have you received at least one dose of a COVID-19 vaccine so far?	Binary (“yes [1]” or “no [0]”).
**Perceptions of COVID-19 infection and booster vaccination**	Self-reported HBM items assessed on a five-point Likert scale, please see Table A2 for detail.	Five-point answers were converted to binary variables, e.g., agree [1] (i.e., “strongly agree” or “agree”) and neutral/disagree [0] (i.e., “neither agree nor disagree”, “disagree” or “strongly disagree”).
**Sociodemographics**	Which age group are you currently in?	Four age groups (18–30, 31–40, 41–50, and 51–59 years old).
	What is your gender?	Binary (“female [1]” or “male [0]”).
	What is your maternal status?	Three groups were converted to binary variables: married [1] (“married”) and never married/divorced/widowed [0] (“never married” or “divorced or widowed”).
	What is your education level?	Four groups were converted to binary variables: senior high school/technical school or below [0] (“middle school or below” or “senior high school/technical school”) and college/associate/bachelor’s degree or above [1] (“college/associate/bachelor’s degree” or “master’s degree or above”).
	What is your employment status?	Four groups were converted to binary variables: employed [1] and retired/out of work/still a student [0] (“retired”, “out of work”, or “still a student”).
	How much is your household annual income?	Three income groups (≤100,000, 100,001–200,000, and >200,000 CNY).
	Which area do you reside in?	Binary (“urban [1]” or “rural [0]”).
	Which province do you reside in?	Respondents were further categorized into living in western, central, and eastern regions based on the province they reside in.
	Rate your own overall health status.	Five-point Likert scale (“very good” to “very poor”). The answers were converted to binary variables: good [1] (“very good” or “good”) and fair/poor [0] (“fair”, “poor”, or “very poor”).
	Do you have any chronic diseases such as hypertension, diabetes, heart disease, etc.?	Binary (“yes [1]” or “no [0]”).
	Do you belong to any of the following priority groups for COVID-19 vaccination? (Health professionals, community workers, workers in the cold-chain logistics sector, customs inspectors, etc.).	Binary (“yes [1]” or “no [0]”).

**Table A2:** Question list of six health belief model dimensions used in this survey.

Dimensions	Questions	Question Design
**Perceived susceptibility**	1. What do you think of the risk of COVID-19 infection?	Five-point Likert scale (“very high” to “very low”)
	2. Do you agree that variants have higher risk of infection than the existing strains?	Five-point Likert scale (“strongly agree” to “strongly disagree”)
**Perceived severity**	1. What do you think of the severity of COVID-19 infection?	Five-point Likert scale (“very high” to “very low”)
	2. Do you agree that variants can cause more severe illness than the existing strains?	Five-point Likert scale (“strongly agree” to “strongly disagree”)
**Perceived benefits**	1. What do you think of the efficacy of boosters against early circulating strains if they become available in the future?	Five-point Likert scale (“very high” to “very low”)
	2. What do you think of the efficacy of boosters to extend protection if they become available in the future?	Five-point Likert scale (“very high” to “very low”)
	3. What do you think of the efficacy of boosters against variants if they become available in the future?	Five-point Likert scale (“very high” to “very low”)
**Perceived barriers**	1. What do you think of the safety of boosters if they become available in the future?	Five-point Likert scale (“very high” to “very low”)
	2. Do you worry about serious adverse reaction after vaccination?	Five-point Likert scale (“very high” to “very low”)
**Self-efficacy**	1. Do you agree that it is easy for you to get the COVID-19 vaccine if you wanted to?	Five-point Likert scale (“strongly agree” to “strongly disagree”)
**Cues to action**	1. Did you use to have confirmed or suspected cases in your daily close contacts?	Binary (“yes” or “no”)
	2. Do you know about the following foreign variants?	Multiple choices of variants
